# Impaired Dynamic Cerebral Autoregulation and Cerebrovascular Reactivity in Middle Cerebral Artery Stenosis

**DOI:** 10.1371/journal.pone.0088232

**Published:** 2014-02-04

**Authors:** Jie Chen, Jia Liu, Wei-Hai Xu, Ren Xu, Bo Hou, Li-Ying Cui, Shan Gao

**Affiliations:** 1 Department of Neurology, Peking Union Medical College Hospital, Chinese Academy of Medical Sciences and Peking Union Medical College, Beijing, China; 2 Shenzhen Institutes of Advanced Technology, Chinese Academy of Sciences, Shenzhen, China; 3 Department of Radiology, Peking Union Medical College Hospital, Chinese Academy of Medical Sciences and Peking Union Medical College, Beijing, China; University Medical Center (UMC) Utrecht, Netherlands

## Abstract

**Purpose:**

We sought to investigate the capacity of cerebral autoregulation and cerebrovascular reactivity (CVR) in patients with middle cerebral artery (MCA) stenosis.

**Methods:**

Twenty-one patients with MCA stenosis diagnosed by magnetic resonance angiography and 15 healthy controls were enrolled. Cerebral autoregulation was assessed by autoregulatory parameters (rate of recovery/phase/gain) derived from transfer function from spontaneous oscillations of cerebral blood flow velocity and blood pressure. CVR was tested by a rebreathing maneuver.

**Results:**

Rate of recovery, phase and CVR estimated from moderate MCA stenosis (rate of recovery  = 17.76±8.21%/s, phase  = 26.93±15.67°, and CVR  = 1.53±0.84%/mmHg, respectively) were significantly different (p<0.05) from controls (rate of recovery  = 39.62±27.99%/s, phase  = 55.66±22.10°, and CVR  = 2.18±0.80%/mmHg, respectively). Rate of recovery (r = −0.698, p<0.001), phase (r = −0.738, p<0.001)) and CVR (r = −0.690, p<0.001) were all significantly correlated with the degree of stenosis.

**Conclusion:**

Cerebral autoregulation and CVR were impaired in patients with ≥ 50% MCA stenosis. The measures of both hemodynamic properties were inversely correlated with the stenotic degree.

## Introduction

Intracranial artery occlusive diseases, especially middle cerebral artery (MCA) stenosis, are major causes of ischemic stroke in Asian, Black, and Hispanic populations [1]. In Chinese populations, they account for approximately 33–50% of stroke and 45% of transient ischemic attack (TIA) [1].

A number of studies reported that stenosis in internal carotid artery (ICA) may impair cerebral hemodynamics, including cerebral autoregulation and cerebrovascular reactivity (CVR), which increases the risk of cerebral ischemic events [2]–[8]. It is however unknown how these cerebral hemodynamic parameters alter with respect to the stenotic degree in MCA. One would expect that both physiological mechanisms become affected interactively, as stenosis is likely to cause vasodilation which reduces CVR and, at the meantime, also decreases the effectiveness of autoregulation. Nevertheless, according to the previous studies on ICA stenosis, autoregulation is much vulnerable to the stenotic changes yet with almost immediate restoration after carotid endarterectomy or stenting, whereas the status of CVR varies in patients and the response to carotid recanalization is not as sensitive as autoregulation [9]. This is sensible as they are intrinsically controlled by different mechanisms. Cerebral autoregulation can be affected by a number of physiological parameters, such as transmural pressure, carbon dioxide (CO_2_), autonomic function, intracranial pressure, and etc., whereas CVR is solely in connection with vasodilation or vasoconstriction. It hence necessary to investigate these two mechanisms in patients with MCA stenosis.

With the advent of transcranial Doppler (TCD) and continuous blood pressure measurement techniques, dynamic cerebral autoregulation, considering short term dynamics between cerebral blood flow velocity (CBFV) and arterial blood pressure (ABP), has been widely studied using transfer function analysis (TFA), providing information both in frequency and time domain [2]–[4], [10]–[14]. We thus assessed dynamic cerebral autoregulation using this technique. Autoregulatory parameters (rate of recovery (RoRc)/phase/gain) can then be derived from TFA to indicate the status of cerebral autoregulation. The test of CVR is rather standardized by inducing hypercapnia and calculating the increment of CBFV with respect to the inspired CO_2_.

Applying TFA, Haubrich et al. demonstrated that the phase shift between ABP and CBFV oscillations were reduced with increasing degree of MCA stenosis compared with controls [15]. Gong et al. also reported the impaired cerebral autoregulation in patients with MCA stenosis, using the parameter of autoregulatory index evaluated using thigh cuff method [16]. Both studies, however, did not exclude patients with either acute stroke or white matter disease (WMD), which may affect the effectiveness of autoregulation [17]–[19]. Cerebral autoregulation may become impaired after stroke, even in the unaffected hemispheres, though it might recover within the next 3 months [17]. And the impaired autoregulation and CVR have also been demonstrated in patients with WMD [18], [19]. The present study excluded these confounding factors (stroke and WMD) and attempt to show if MCA stenosis is an independent factor affecting cerebral autoregulation and CVR and how these hemodynamic parameters change with respect to the degree of stenosis in MCA.

## Methods

### Subjects

The study was approved by the Peking Union Medical College Hospital Research Ethics Committee and each participant gave written informed consent.

Twenty-one patients with stenosis in M1 segment of MCA diagnosed by magnetic resonance angiography were recruited from March to April, 2012. Patients were excluded from the study, if they had coexistent ≥50% ipsilateral ICA stenosis, stroke or TIA onset within 3 months, or severe WMD (≥3 grades for either periventricular hyperintensity or deep white matter hyperintensity based on the Fazekas visual scale) [20]. All patients received careful evaluations of vascular risk factors and history of stroke.

Fifteen age-matched healthy individuals were recruited as control subjects. Controls who were found to have carotid artery and intracranial stenosis by TCD were excluded [21].

### Image Acquisition, Degree of Stenosis, and WMD Severity Scales

All patients were imaged using a 3T scanner (Signa VH/i, GE Medical Systems, Milwaukee, WI, USA). The typical magnetic resonance image (MRI) protocol included at least T1 and T2-weighted images, fluid- attenuated inversion recovery (FLAIR), and 3-dimensional time-of-flight magnetic resonance angiography (MRA). The FLAIR parameters were as follows: repetition time/echo time, 8000/165 ms; number of excitations, 1; flip angle, 90°; field of view, 24 cm × 24 cm; matrix size, 288×192; slice thickness, 6 mm; and slice gap, 1 mm. MRA was performed using the following parameters: repetition time/echo time, 27/6.9 ms; number of excitations, 1; flip angle, 20°; field of view, 24 cm × 16 cm; matrix size, 320×256; and slice thickness, 1.6 mm.

The degree of MCA stenosis was graded as mild (<50%), moderate (50%–69%), or severe stenosis (70%–99%, but with apparent distal segment) [22].

The severity of white matter lesion was assessed by using FLAIR images, except for one patient using T2-weighted images, whose motion artifact was severe on FLAIR. Both the periventricular hyperintensity and deep white matter hyperintensity of each side were graded into 3 stages (grade 0-I, grade II, grade III) by a radiologist, according to the Fazekas rating scale [20].

### Measurement of Dynamic Autoregulation and Cerebrovascular Reactivity

**Figure 1:**
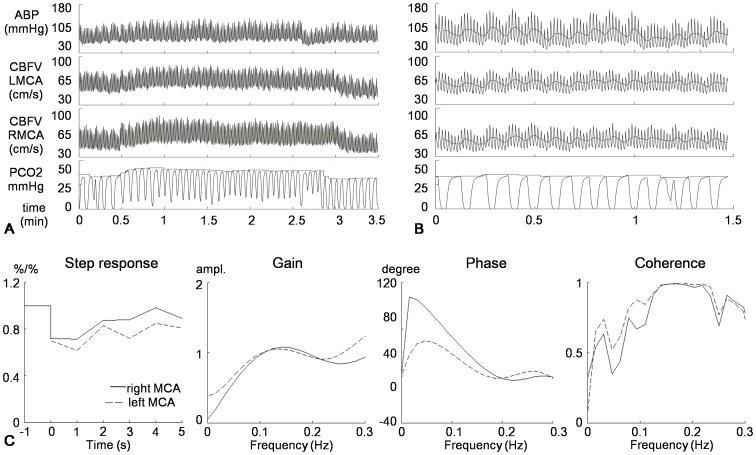
Illustrative recording of cerebrovascular reactivity testing and dynamic cerebral autoregulation analysis. A 52-yr-old patient with severe stenosis of the left MCA and mild stenosis of the right MCA. (A) A rebreathing test for cerebrovascular reactivity. Values were 1.30%/mmHg on the left and 2.21%/mmHg on the right side. (B) Raw data of spontaneously breathing, showing the oscillations of the blood pressure and cerebral blood flow velocity. (C) Transfer function analysis shows, compared with the right side, the recovery of the blood flow of the left side was slower, the phase shift was lower, and the coherence was higher.

Bilateral flow velocity of MCAs was measured via TCD (mutidop-X2, DWL, Sipplingen, Germany) by using 2 MHz transducers fixed to the head with the headframe (Spencer Technologies, Seattle, WA, USA). The distal segment of the affected MCA was insonated, at the depth of 45 to 50 mm. Continuous beat-to-beat ABP was registered by a servo-controlled finger plethysmograph (Nexfin-cc, BMEYE, Amsterdam, Netherlands) in the right hand positioned at the heart level. End-tidal partial pressure of carbon dioxide (PETCO_2_) was measured with an infrared capnometer (OEM-Module, Envitec, Wismar, Germany) connected via a breathing mask cover the nose during nasal expiration. All the subjects breathed spontaneously in supine position. After a resting time of 10 minutes, the CBFV of bilateral MCAs, ABP, and PETCO_2_ were recorded for at least 5 minutes. And then a half blocked long tube (1 meter, 600 mL) was connected with the mask for 2 minutes, which composed a semi-closed system for the accumulation of exhaled CO_2_ [23]. With the increasing of the concentration of CO_2_ in the semi-closed tube, the CO_2_ inhalation of the subject was increased (Fig. 1). All the data (including CBFV, ABP, and PETCO_2_) were recorded online in a sample rate of 100 Hz and stored on a hard disk for further analysis.

### Autoregulation Analysis

The dynamic cerebral autoregulation was evaluated using TFA [12], [13]. The recorded ABP and CBFV were filtered by an anti-aliasing filter (a 3rd order Butterworth low-pass filter) with cut-off frequency at 0.5 Hz and then down-sampled to 1 Hz. The auto-spectrum, 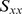, of ABP and the oss-spectrum, 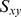, between ABP and CBFV were estimated using Welch's method, a modified periodogram method. The transfer function was then calculated as:
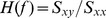
(1)


Phase and gain were then computed as:
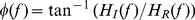
(2)

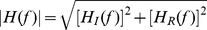
(3)where 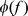 is the phase response and 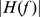 is the gain of the transfer function, respectively. 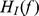 and 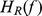 denote the image and real part of the transfer function, respectively.

The magnitude squared coherence function, 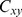, was then estimated also using Welch's method. The coherence function is a function of frequency with values between 0 to 1, indicating how well ABP corresponds to CBFV in frequency, which is formulated as:
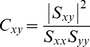
(4)where 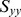 is the auto-spectrum of CBFV. The impulse and frequency responses can then be derived from 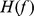 by inverse Fourier transform. The step response of CBFV can then be calculated by the convolution between the impulse response and a normalized step change of ABP from 1 to 0, which shows the recovery of cerebral blood flow after a step-wise change of ABP. In order to quantify the speed of the recovery, we applied a previously defined parameter, the RoRc of CBFV (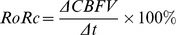), where the first 3 seconds of the step response were used for the calculation [14]. The step response was normalized by the first value to reduce the effect of under- or overshoot of the step response. In frequency domain, we only used the parameters (phase, gain, and coherence function) within 0.06–0.12 Hz to evaluate cerebral autoregulation.

### Cerebrovascular reactivity Analysis

The CVR was calculated as the maximum percentage increase of CBFV during hypercapnia per unit increase in PETCO_2_ (in mmHg) [5].

### Statistical Analysis

In statistical analysis, Student's t-test, Analysis of variance (ANOVA) and SNK-q test were performed to compare the continuous variables across groups, and Pearson χ^2^ was used to compare categorical variables between groups. The correlation between autoregulatory parameters and degree of MCA stenosis, was analyzed by Spearman's method. The correlation between phase shift and CVR was analyzed by Pearson's method. A p value less than 0.05 was considered statistically significant. All statistical analysis was done with SPSS 16.0 (SPSS Inc, Chicago, IL, USA).

## Results

Among all the 21 patients recruited, six could be classified as symptomatic (four experienced ischemic stroke and two had TIA). More patients had history of hypertension compared with controls, but there were no significant difference between cases and controls in age, sex, history of diabetes, and previous or current smoking (Table 1).

**Table 1:** Clinical characteristics of subjects with MCA stenosis and controls.

	MCA stenosis	Controls	*P*
	(n = 21)	(n = 15)	
Age, years	56.72±15.24	49.8±11.88	0.152
Female, n(%)	13 (61.9)	9 (60.0)	0.908
Hypertension, n (%)	14 (66.7)	3 (20.0)	0.028
Diabetes mellitus, n (%)	4 (19.0)	3 (20.0)	0.943
previous or current smoking, n (%)	7 (33.3)	7 (46.7)	0.418

MCA, middle cerebral artery.

All subjects were examined bilaterally except for 2 patients with inadequate temporal window of the unaffected side. Thus, a total of 70 MCAs were investigated including 32 stenotic MCAs and 38 normal MCAs (bilateral stenosis was found in 11 patients). The distribution of the 32 stenotic MCAs was as follows: mild stenosis, 8 hemispheres; moderate stenosis, 11 hemispheres; and severe stenosis, 13 hemispheres.

The average PETCO_2_ was 33.10±5.06 mmHg during normocapnia, and 46.21±4.15 mmHg during hypercapnia. And the rebreathing procedure induced an increase of ABP about 3.98±4.23 mmHg.

**Figure 2:**
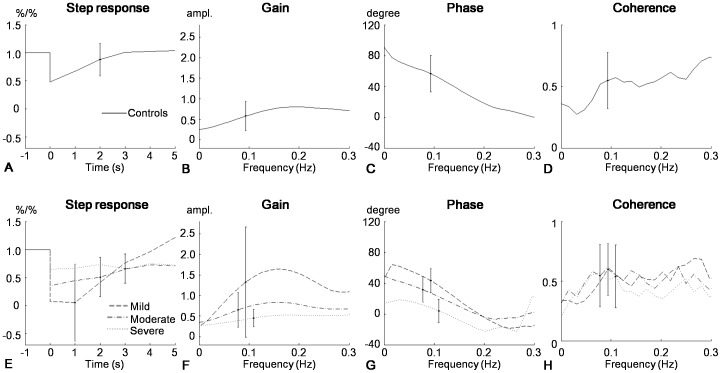
Time and frequency domain analysis of cerebral autoregulation using transfer function. The figures in rows are the averaged step response, gain, phase, and coherence function, respectively for the controls (**A–D**) and patients with middle cerebral artery stenosis (**E–H**), derived from the transfer function. As defined in (**E**), different line styles in the patients' row denote the stages, where mild, moderate, severe stenosis are the dashed, dashed-dotted, dotted, respectively. Each vertical bar shows the averaged standard deviation of the plotted parameter.

**Figure 3:**
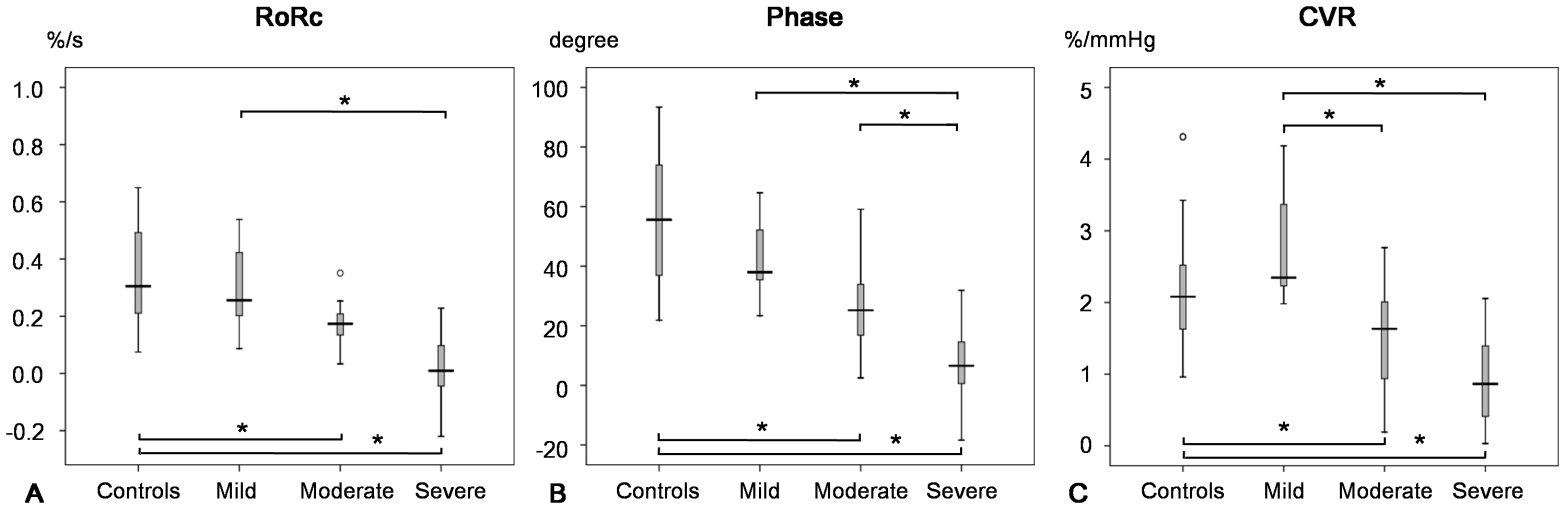
Box plots of cerebral autoregulation and cerebrovascular reactivity (CVR). The figures in rows are the rate of recovery (RoRc) (**A**), phase (**B**), and CVR (**C**) according to the degree of middle cerebral artery (MCA) stenosis. * denotes p<0.05 between the groups. ○ denotes exceeding values (>1.5 box length).

Time and frequency analysis of cerebral autoregulation using transfer function were shown in Fig. 1 and the corresponded statistical analysis was given in Table 2 and Fig. 2. In the time domain, the averaged step response estimated from the controls was evidently steeper than hemispheres with mild stenosis (Fig. 2A,E). The step response could rapidly reach the baseline level within approximately 3 seconds for the controls, whilst it took 4 seconds for the hemispheres with mild stenosis, indicating a slower recovery of blood flow for the mild stenotic hemispheres, if ABP changed in a step-wise manner. The situation became even worse as stenosis develops, where, in moderate to severe stenosis, the averaged step responses were almost flat, implying cerebral blood flow could not be regulated to the baseline level within 5 seconds. The autoregulatory parameter, RoRc, showed a trend towards poorer value, but only moderate to severe stenosis reached statistic significance (Fig. 3A). The decrease of the RoRc was significantly correlated with the degree of stenosis (correlation coefficients  = −0.70, p<0.001).

**Table 2:** Results of RoRc, phase shift, magnitude, coherence and CVR in controls and different degree of MCA stenosis.

		Degree of MCA Stenosis		
	Controls	Mild	Moderate	Severe
	(n = 38)	(n = 8)	(n = 11)	(n = 13)
RoRc(%/s)	39.62±27.99	29.81±16.10	17.76±8.21*	1.16±13.22*
Gain (ampl.)	0.58±0.35	1.30±1.34*	0.70±0.42	0.42±0.20
Phase (degree)	55.66±22.10	42.37±13.10	26.93±15.67*	6.88±14.14*
Coherence	0.51±0.18	0.51±0.15	0.56±0.20	0.52±0.23
CVR(%/mmHg)	2.18±0.80	2.76±0.85	1.53±0.84*	0.98±0.67*

MCA, middle cerebral artery; RoRc, rate of recovery, CVR: cerebrovascular reactivity;

* p<0.05, compared with normal controls.

In the frequency domain, the gain estimated from the controls and the patients all showed the shape of a high-pass filter, suggesting autoregulation was active (Fig. 2B). However, the shape showed a trend towards flat from mild to severe stenosis (Fig. 2F). The mean values (see Table 2) of gain at 0.06–0.12 Hz was decreased gradually when the stenosis became more severe, which was significantly correlated (correlation coefficients  = −0.41, p = 0.02) with the degree of stenosis. In Fig. 1C, D, we observed that the phase at 0.06–0.12 Hz reduced as stenosis towards severe degree. ANOVA indicated that compared with controls, MCAs with moderate to severe stenosis showed pronounced reduction of phase shift (Fig. 3B). And Decrement of Phase shift was highly correlated with the degree of stenosis (correlation coefficient  = −0.74, p<0.001). But there was no significant difference between cases and controls in coherence (Table 2).

With respect to CVR, the value was also significantly reduced in hemispheres with moderate to severe stenosis compared with controls (Table 2, Fig. 3C). The decrement of CVR was well correlated with the degree of stenosis (correlation coefficient  = −0.69, p<0.001).

The CVR was significantly correlated with phase shift and RoRc, with the correlation coefficient of 0.44 and 0.38 (p<0.001), pooling all hemisphere (n = 71).

## Discussion

In general, the autoregulatory parameters (phase/RoRc) derived from transfer function showed that the effectiveness of cerebral autoregulation is inversely correlated with the degree of stenosis. CVR is also significantly decreased with respect to the degree of stenosis. We also found that the measures of both mechanisms were significantly correlated. These major findings are in line with previous studies on cerebral autoregulation and CVR [15], [16], [23].

It is however worth noting that the transfer function gain estimated in the patients with mild stenosis is higher than that in the controls. Though it is expected that impaired autoregulation results in higher gain as it indicates a less dampening effect, this finding, however, disagrees with previous studies on ICA and MCA stenosis [3], [11], [15]. One possible reason for this is that we defined mild stenosis with lower degree of stenosis (<50%) which may merely cause subtle dilation of arterioles, allowing more passive changes of CBFV in amplitude with respect to the changes of ABP. However, in the previous studies, authors reported decreased gain when mild stenosis was defined with higher degree of stenosis [3], [11], [15]. The decreased gain in their studies was attributed to the inability to achieve active diameter changes in fully (or almost fully) dilated arterioles, resulting in decreased CBFV amplitude oscillations [3], [11].The higher gain also led to amplified changes of the step response when comparing with other stages. Furthermore, this can also explain the slightly ‘better’ CVR than the controls as we observed in Fig. 3C, as impaired autoregulation may trigger ‘passive autoregulation’ due to a possible rise in ABP associated with inspiration of increased levels of inspired CO_2_ [24]. However, further investigation on this is required to confirm.

We found significant but relatively low correlation coefficients between CVR and the autoregulatory parameters – the phase shift and RoRc (0.44 and 0.38 for phase and RoRc, respectively). This is in consistent with previous study in ICA stenosis [2]. We consider that the relatively low correlation might indicate that the impairment of cerebral autoregulation is only partially attributed to the vasodilation caused by stenosis, as cerebral autoregulation and CVR are intrinsically two different mechanisms [25], [26]. Indeed, we did observe evident increase of CBFV and ABP (more than 10 mmHg increment in ABP in two cases) in response to the rebreathing test, which imposes a multivariate process of the hemodynamics. The current TFA assuming cerebral autoregulation as a univariate process may therefore fail to interpret it. Multivariate modeling techniques developed by a number of recent studies might be helpful in the future study of this interactive dynamics [27]–[30].

Although artery-to-artery embolism is considered as the major mechanism causing ischemia in intracranial atherosclerotic disease, hypo-perfusion with impaired embolism clearance may also play a role [31]. In patients with intracranial occlusive disease, only pial or menigeal collaterals are available to maintain the cerebral perfusion pressure [32]. When collaterals are not adequate, decreases in perfusion pressure may lead to vasodilation of cerebral arterioles and potential impairment of cerebral reserve capacity. Using TCD to test CVR is a non-invasive and non-pharmacologic technique to access the cerebral reserve capacity after intracranial stenosis. And our study showed that both autoregulation and CVR reduction are linearly correlated with degree of MCA stenosis. Thus the assessment of these two hemodynamic mechanisms might thus be helpful in monitoring the progress of MCA stenosis.

According to our results, cerebral autoregulation was significantly impaired in patients with moderate to severe MCA stenosis. Therefore, it should be cautious when considering antihypertensive therapy for these patients, as cerebral blood flow may become ‘vulnerable’ to the changes of ABP. For instance, drops in ABP might result in hypo-perfusion, that may impair the clearance of emboli and thereby increase the risk of ischemia. Thus assessment of cerebral hemodynamics in patients with intracranial stenosis should be considered, especially for the guidance of hypertensive therapy.

In summary, this study shows that cerebral autoregulation and CVR are impaired in patients with moderate to severe MCA stenosis. Furthermore, with increasing degree of stenosis, the impairment of autoregulation and CVR tend to be worsened. The assessment of these two hemodynamic mechanisms might thus be helpful in monitoring the progress of MCA stenosis.
